# β-Galactosylceramidase Deficiency Causes Bone Marrow Vascular Defects in an Animal Model of Krabbe Disease

**DOI:** 10.3390/ijms21010251

**Published:** 2019-12-30

**Authors:** Mirella Belleri, Daniela Coltrini, Marco Righi, Cosetta Ravelli, Sara Taranto, Paola Chiodelli, Stefania Mitola, Marco Presta, Arianna Giacomini

**Affiliations:** 1 Department of Molecular and Translational Medicine, University of Brescia, 25123 Brescia, Italy; mirella.belleri@unibs.it (M.B.); daniela.coltrini@unibs.it (D.C.); cosetta.ravelli@unibs.it (C.R.); sara.taranto@unibs.it (S.T.); paola.chiodelli@unibs.it (P.C.); stefania.mitola@unibs.it (S.M.); 2 Institute of Neuroscience, National Research Council, 20129 Milan, Italy; m.righi@in.cnr.it; 3 Unity of Brescia, Italian Consortium for Biotechnology, 25123 Brescia, Italy

**Keywords:** bone marrow, endothelium, beta-galactosylceramidase, Krabbe disease, *twitcher* mice

## Abstract

Krabbe disease (KD) is an autosomal recessive sphingolipidosis caused by the deficiency of the lysosomal hydrolase β-galactosylceramidase (GALC). Oligodendroglia degeneration and demyelination of the nervous system lead to neurological dysfunctions which are usually lethal by two years of age. At present, the only clinical treatment with any proven efficacy is hematopoietic stem-cell transplantation, which is more effective when administered in the neonatal period to presymptomatic recipients. Bone marrow (BM) sinusoidal endothelial cells (SECs) play a pivotal role in stem cell engraftment and reconstitution of hematopoiesis. Previous observations had shown significant alterations of microvascular endothelial cells in the brain of KD patients and in *Galc* mutant *twitcher* mice, an authentic model of the disease. In the present study, we investigated the vascular component of the BM in the femurs of symptomatic homozygous *twitcher* mice at postnatal day P36. Histological, immunohistochemical, and two-photon microscopy imaging analyses revealed the presence of significant alterations of the diaphyseal BM vasculature, characterized by enlarged, discontinuous, and hemorrhagic SECs that express the endothelial marker vascular endothelial growth factor receptor-2 (VEGFR2) but lack platelet/endothelial cell adhesion molecule-1 (CD31) expression. In addition, computer-aided image analysis indicates that *twitcher* CD31^−^/VEGFR2^+^ SECs show a significant increase in lumen size and in the number and size of endothelial gaps compared to BM SECs of wild type littermates. These results suggest that morphofunctional defects in the BM vascular niche may contribute to the limited therapeutic efficacy of hematopoietic stem-cell transplantation in KD patients at symptomatic stages of the disease.

## 1. Introduction

Lysosomal storage disorders are characterized by the accumulation of disease-specific metabolic intermediates within the lysosomes [1]. Krabbe disease (KD), or globoid cell leukodystrophy, is an autosomal recessive sphingolipidosis caused by the deficiency of the lysosomal hydrolase *β-galactosylceramidase* (GALC) (EC 3.2.1.46) [2]. The disease is characterized by degeneration of oligodendroglia and progressive demyelination of the peripheral and central nervous system (CNS). GALC degrades galactosylceramide and other terminal β-galactose-containing sphingolipids, including galactosylsphingosine (psychosine). The pathogenesis of the disease has been proposed to arise from the accumulation of this neurotoxic metabolite present at high levels in the CNS of Krabbe patients [1,3,4].

Clinically, KD manifests in early infancy with fatal neurological dysfunctions [5,6,7]. The current standard of care for this disease is hematopoietic stem cell transplantation (HSCT) derived from bone marrow (BM) or umbilical cord blood [7,8]. Clinical studies report long-term functional outcomes in presymptomatic KD infants who undergo HSCT in the first month of life, whereas the progression of the disease is not reversed by HSCT when performed after symptom onset [9,10]. In addition, a study using *Galc* mutant *twitcher* mice, an authentic animal model of KD [11,12], has reported a lower rate of engraftment in homozygous *Galc*^−/−^ recipients compared to heterozygous *Galc*^−/+^ mice [13], pointing to the presence of possible defects in the hematopoietic stem cell niche of GALC deficient mice.

The BM hematopoietic stem cell (HSC) niche provides soluble factors, forces, and cell-mediated interactions necessary to maintain the hematopoietic potential of the stem cells and to allow the process of hematopoiesis [14]. This niche is composed by the endosteal niche (containing osteocytes, bone matrix, and quiescent HSCs) and the perivascular niche (containing actively dividing HSCs, reticular cells, mesenchymal stem cells, and sinusoidal endothelial cells (SECs)) [14]. HSCs strongly interact with BM vasculature after HSCT and this interaction is important for stem cell engraftment and reconstitution of hematopoiesis [15]. In particular, functional BM SECs determine the degree of hematopoietic recovery being essential for HSC engraftment and hematopoiesis [15]. Altogether, these findings suggest that vascular alterations in the BM vascular niche of symptomatic Krabbe patients might affect HSC engraftment after transplantation.

The effect of GALC deficiency on CNS microvascularization and the angiogenic process has been previously investigated [16,17,18,19]. GALC deficiency, with consequent psychosine accumulation, induces ultrastructural and functional defects in the endothelium of the brain of *twitcher* mice. Accordingly, significant alterations have been observed in human cortex microvasculature from brain biopsy of a KD patient [19]. These studies also identified psychosine as an endothelial actin-disassembling agent endowed with antiangiogenic activity in vitro and in vivo [19]. Notably, GALC deficiency causes psychosine accumulation also in non-nervous tissues/organs, including liver, kidney, and lungs [20,21]. Indeed, GALC deficiency in *twitcher* mice is responsible for postnatal bone growth retardation [22], liver damage [23], and lymphoid organ atrophy [24], supporting the notion that KD is a generalized psychosine storage disease [20]. In keeping with this hypothesis, significant vascular permeability defects occur in different visceral organs of *twitcher* mice, including kidney, lung, and liver [19]. Thus, a systemic endothelium-related pathogenic aspect of KD may exist. This may contribute to worsening the disease evolution and may adversely affect therapeutic interventions, including BM repopulation following HSCT. 

On this basis, in the present study we investigated the vascular component of the BM of *twitcher* mice. The results reveal the presence of significant alterations in BM SECs, in keeping with the hypothesis that defects in the vascular niche may contribute to the limited therapeutic efficacy of HSCT in KD patients at symptomatic stages of the disease.

## 2. Results

**Figure 1:**
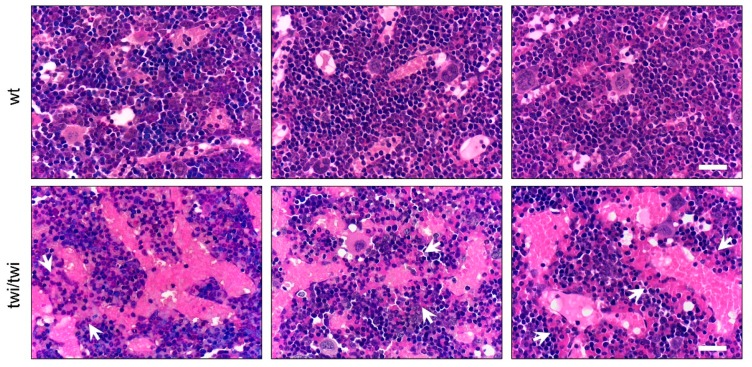
Abnormal BM vasculature in *twitcher* mice. H&E staining of femurs from three wt and three *twitcher* (twi/twi) mice. White arrows indicate hemorrhagic areas. Scale bar: 50 μm.

To analyze the vascular component of the BM of *twitcher* (twi/twi) mice, we first performed a histological analysis of femurs from littermate wild type (wt) and symptomatic homozygous twi/twi animals at postnatal day P36. Hematoxylin and eosin (H&E) staining revealed the presence of abnormal SECs in BM sections from *twitcher* mice compared to wt animals. Indeed, the vasculature of femurs from wt animals resulted composed by thin and regularly distributed SECs whereas the BM vasculature from twi/twi mice was characterized by enlarged, discontinuous, and hemorrhagic SECs brimful of red blood cells (Figure 1).

**Figure 2:**
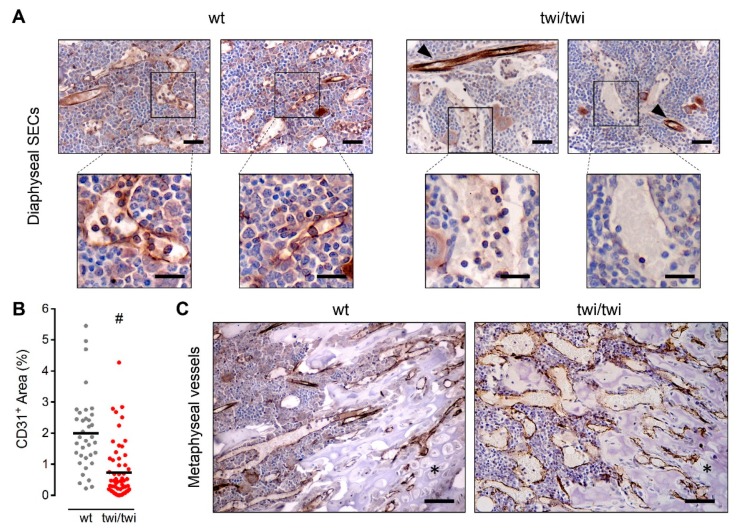
BM diaphyseal *twitcher* SECs do not express CD31. CD31 immunohistochemical staining of femurs from wt and *twitcher* mice. (**A**) CD31 expression in diaphyseal SECs of two wt and two *twitcher* mice. Arrowheads indicate CD31^+^ arterioles. Scale bar: 50 μm. Scale bar in magnified fields: 30 μm. (**B**) Quantification of CD31^+^ area in femoral histological sections. Dots represent the percentage area occupied by CD31^+^ vessels in different microscopic fields. Horizontal black bars show the mean value. #, *p* < 0.001. (**C**) CD31 expression in metaphyseal vessels. Asterisks indicate the cartilaginous tissue. Scale bar: 100 μm.

In order to get further insights into the vascular component of *twitcher* BM, we performed immunohistochemical staining of the endothelial markers platelet/endothelial cell adhesion molecule-1 (PECAM-1/CD31) and vascular endothelial growth factor receptor-2 (VEGFR2) that are expressed by both BM arterioles and capillaries [15]. Interestingly, immunohistochemical analysis revealed the complete absence of CD31 expression by abnormal SECs in *twitcher* mice, which was instead maintained in arteriolar endothelium (Figure 2A).

Accordingly, quantification of CD31^+^ signal in BM sections showed a strong reduction of CD31 expression in twi/twi versus wt femurs (Figure 2B). Notably, the lack of CD31 immunoreactivity appeared to be limited to diaphyseal SECs whereas CD31 expression was detectable in methaphyseal *twitcher* vessels (Figure 2C).

**Figure 3:**
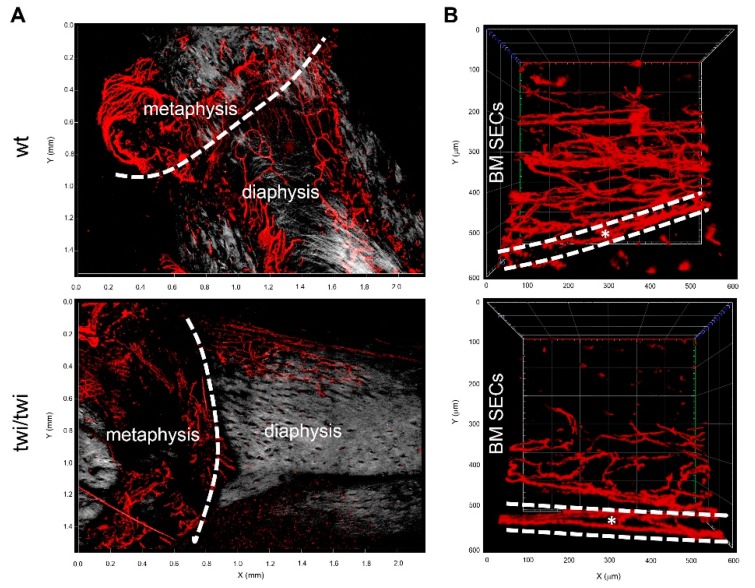
Two-photon microscopy analysis of CD31 expression in diaphyseal, metaphyseal and cortical bone vessels of *twitcher* mice. (**A**) 3D reconstruction of CD31^+^ vessels (in red) and stromal collagen expression revealed by second harmonic generation (in grey) in diaphysis and metaphysis of wt and *twitcher* mice (Z = 422 μm and 588 μm for wt and twi/twi specimens, respectively). (**B**) Cross-sectional view of CD31^+^ vessels in diaphysis and cortical bone (dashed white lines) of wt and *twitcher* mice (Z = 294 μm and 352 μm, respectively).

Two-photon microscopy imaging performed on clarified femurs of *twitcher* mice confirmed the lack of CD31 expression in diaphyseal SECs whereas endothelial CD31 expression was maintained in cortical bone and methaphyseal vessels of the *twitcher* femurs (Figure 3).

**Figure 4:**
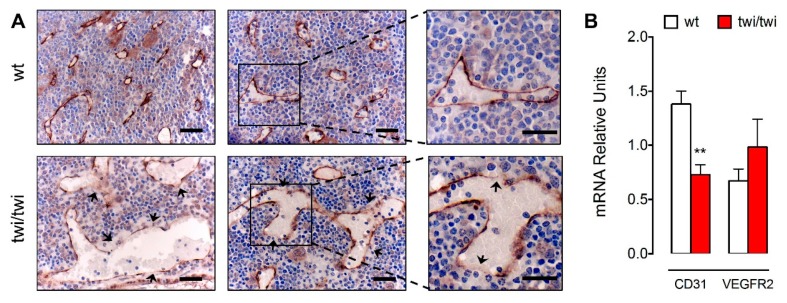
VEGFR2 is expressed by the BM vasculature of *twitcher* mice. (**A**) VEGFR2 immunohistochemical staining of femoral sections from two wt and two *twitcher* mice. Arrows indicate endothelial gaps. Scale bar: 50 μm. Scale bar in magnified fields: 30 μm. (**B**) *Cd31* and *Vegfr2* mRNA expression levels in wt and *twitcher* BM as assessed by RT-qPCR analysis. Data are mean ± SEM of 7–8 animals per group. **, *p* < 0.01.

At variance with CD31, immunohistochemical analysis showed that VEGFR2 was equally expressed in SECs of twi/twi and wt BM (Figure 4A).

In keeping with what observed in H&E-stained sections, abnormal CD31^−^/VEGFR2^+^ SECs form enlarged vessels in *twitcher* femurs, characterized by the presence of numerous endothelial gaps. Reduced levels of CD31 and unaltered levels of VEGFR2 expression in twi/twi *versus* wt BM were confirmed also by quantitative RT-PCR (RT-qPCR) analysis of tissue samples obtained after femur flushing (Figure 4B).

**Figure 5:**
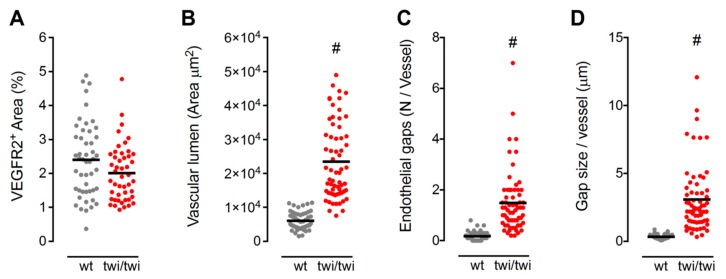
Quantification of BM vascular abnormalities in *twitcher* mice. (**A**) Quantification of the BM area occupied by VEGFR2^+^ diaphyseal SECs. Each dot represents the percentage VEGFR2^+^ area per microscopic field. (**B**–**D**) Morphometric parameters of VEGFR2^+^ diaphyseal SECs, including area of the vessel lumen (**B**), number of endothelial gaps per vessel (**C**), and size of endothelial gaps (**D**). For each parameter, dots represent the mean value measured in different microscopic fields. Horizontal black bars show the mean value. #, *p* < 0.001.

To obtain an objective quantification of the morphological abnormalities of SECs in *twitcher* BM, a computer-aided image analysis was performed on VEGFR2-immunostained femurs from wt and twi/twi mice. This analysis allowed the quantification of the total VEGFR2^+^ vascular area, the vascular lumen size, the number of gaps per vessel and the size of endothelial gaps in the two groups of animals. In keeping with RT-qPCR data, no difference in the total VEGFR2^+^ vascular area was observed in twi/twi versus wt BM (Figure 5A). However, the vessels of *twitcher* BM showed a 4-fold increase in lumen size (Figure 5B) and an 8-fold increase in the number of endothelial gaps per vessel (Figure 5C) compared to wt vessels. In addition, in keeping with the presence of diffuse hemorrhagic areas, the size of these vascular gaps was 9-fold larger in *twitcher* BM compared to wt SECs (Figure 5D).

**Figure 6:**
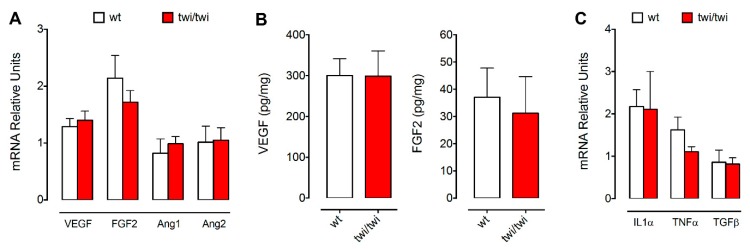
Quantification of pro-angiogenic and pro-inflammatory cytokines in the BM of *twitcher* mice. Pro-angiogenic (**A**,**B**) and pro-inflammatory cytokines (**C**) were quantified after BM flushing by ELISA (**B**; 3 animals per group) and RT-qPCR (**A**,**C**; 7–8 animals per group). Data are mean ± SEM.

Overexpression of pro-angiogenic factors may lead to the formation of abnormal vessels [25]. Thus, in the attempt to understand the causes of the vascular abnormalities observed in the BM of *twitcher* mice, we assessed the expression levels of various pro-angiogenic factors in tissue samples obtained after femur flushing. As shown in Figure 6A, RT-qPCR analysis showed no significant differences in the expression levels of *Vegfa*, *Fgf2*, *Ang1*, and *Ang2* between wt and twi/twi BM samples. Accordingly, wt and twi/twi BM contain similar levels of VEGF-A and FGF2 protein (Figure 6B). We then investigated for the presence of a possible psychosine-induced inflammatory process that may occur in the BM of *twitcher* mice, as described for the brain of these animals [26]. To this aim, we measured the levels of expression of the pro-inflammatory cytokines *Il1α*, *Tnfα* and *Tgfβ*. RT-qPCR analysis revealed no significant changes of the steady-state mRNA levels of these cytokines in the BM of the two groups of animals (Figure 6B), in keeping with previous observations showing unaltered levels of TNFα and IL-6 proteins in the femur of *twitcher* mice [22]. Together, these data rule out a possible involvement of inflammation in determining the BM vascular abnormalities observed in *twitcher* mice.

**Figure 7:**
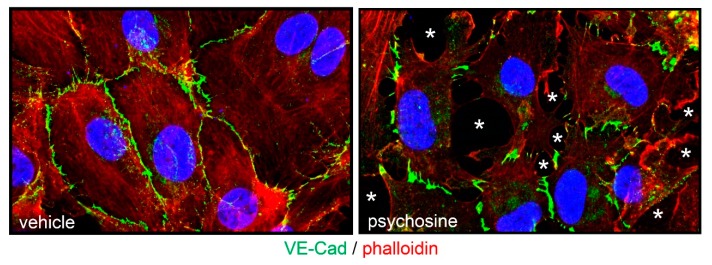
Psychosine affects the endothelial barrier integrity. HUVEC monolayer were treated overnight with 50 μM psychosine and immunostained with an anti-VE-Cad antibody (in green). Actin cytoskeleton was visualized by incubation with rhodamine phalloidin (in red) and nuclei were counterstained with DAPI (in blue). Note the loss of junctional protein organization and the appearance of endothelial gaps (asterisks) in the psychosine-treated HUVEC monolayer.

Previous observations had shown that psychosine exerts an anti-angiogenic activity on endothelial cells by hampering their cytoskeleton organization, proliferation, migration and response to growth factors [19]. On this basis, we evaluated the effect of psychosine administration to a monolayer of human umbilical vein endothelial cells (HUVECs). In keeping with the alterations of the SEC barrier integrity observed in the BM of *twitcher* mice, the appearance of intercellular gaps and the loss of cell contact organization of the vascular endothelial-cadherin (VE-Cad) junction protein characterize HUVEC monolayers treated with psychosine (Figure 7).

## 3. Discussion

For several years, the clinical and pathological manifestations of KD have been almost exclusively restricted to the nervous system because of the accumulation of the neurotoxic GALC substrate psychosine at high levels in the brain and peripheral nerves [1,3,4]. More recently, novel observations have raised the hypothesis that KD may represent a more generalized psychosine storage disease. Indeed, psychosine accumulation has been detected also in non-nervous organs, including liver, kidney, lungs, and bones among others, even though at concentrations lower than those measured in the CNS [20,21]. 

Previous observations from our laboratory had demonstrated that GALC deficiency induces significant endothelial alterations in the microvasculature of brain cortex and non-nervous tissues of *twitcher* mice, an authentic model of the disease [16,17,18,19]. In the present study, the vascular component of the BM of homozygous *twitcher* mice was investigated for the first time. Histological, immunohistochemical and two-photon microscopy imaging analyses have revealed the presence of significant microvascular alterations of the twi/twi BM that were limited to diaphyseal SECs. Indeed, when compared to wild type littermates, *twitcher* diaphyseal SECs formed enlarged, discontinuous and hemorrhagic vessels expressing the endothelial marker VEGFR2 but lacking CD31 expression. At variance with what observed for the microvascular component of *twitcher* brain [16,17,18,19], these alterations occurred in the absence of a significant reduction of the BM vascular density. Thus, the alterations detected in GALC deficient diaphyseal microvasculature resemble, at least in part, those reported for the visceral organs of *twitcher* mice, including liver, kidney, and lungs, characterized by increased vascular permeability with no alterations of their vascular density [19].

Previous observations had shown the ability of psychosine to affect actin reorganization, leading to inhibition of cytokinesis and formation of multinuclear globoid cells, a marker of KD [27,28,29]. Accordingly, psychosine causes the disorganization of actin stress fibers in endothelial cells, leading to significant alterations of the microvascular angioarchitecture [19]. In keeping with the morphological abnormalities that occur in SECs of *twitcher* BM, our data demonstrate that psychosine can cause profound alterations of the barrier integrity of a HUVEC monolayer by increasing the number of intercellular gaps and disrupting VE-Cad organization. Even though psychosine is present in the femurs of *twitcher* mice at levels lower than those occurring in the brain of these animals [22], a long-lasting exposure to this GALC metabolite dramatically increases its capacity to affect endothelial cell functions at concentrations that are compatible with those measured in GALC-deficient non-nervous tissues/organs [19]. This suggests that the alterations occurring in BM SECs might be the consequence of a chronic exposition to low concentrations of psychosine during the postnatal life of *twitcher* mice.

When compared to wild type animals, *twitcher* BM SECs fail to express the endothelial marker CD31. Under normal conditions, BM arterioles and capillaries, including SECs, express CD31 [15]. CD31 is the most abundant component of endothelial cell junctions and plays a pivotal role in maintaining and restoring the vascular permeability barrier [30]. Thus, the lack of CD31 expression, together with the effect of psychosine on endothelial barrier integrity, might contribute to the vascular permeability defects observed in *twitcher* BM SECs. CD31 has been defined as a marker for mature endothelial cells [31]. The absence of CD31 expression by *twitcher* BM SECs might suggest the presence of immature endothelial cells composing the sinusoidal capillary network. On the other hand, loss of endothelial cell-specific proteins, including CD31, has been associated with endothelial to mesenchymal transition, a newly recognized type of cellular transdifferentiation [32,33].

Notably, at variance with diaphyseal SECs, no vascular alterations were observed in epiphyseal/metaphyseal vessels. Angiogenesis is the main mechanism of vessel formation in the bone and it occurs with a different timing in the diaphysis and metaphysis/epiphysis during murine development [34]. The diaphyseal vasculature is formed first, starting at E15, then the metaphyseal and epiphyseal vessels are generated starting at P6 and bone vessel formation is completed at P21 [34]. Thus, the different origin, timing of formation, and microenvironment milieu of diaphyseal versus metaphyseal/epiphyseal vasculature might explain their different response to GALC deficiency. These observations set the basis for future studies leading to a better understanding of the pathogenesis of the alterations that occur in BM diaphyseal SEC of *twitcher* femurs.

GALC deficiency has been associated with impairment of the hematopoietic stem cell niche [13]. Indeed, clinical studies report long-term functional outcomes following HSCT in presymptomatic KD infants, whereas the progression of the disease is not reversed when the transplantation is performed after symptom onset [9,10]. Functional and well-structured BM SECs are essential for HSC engraftment and hematopoiesis [15]. Here, we report for the first time that significant alterations occur in BM SECs of symptomatic *twitcher* mice at P36, suggesting that such alterations may contribute to HSCT failure in symptomatic KD patients. In addition, our observations may explain the decrease in BM and blood cellularity observed in symptomatic *twitcher* mice, characterized by a reduced number of BM B220^+^ B-lymphocytes, SCA-1^+^ hematopoietic stem cells and GR-1^+^ granulocytes paralleled by a normal number of Ter119^+^ erythroid progenitors and peripheral red blood cells [24]. These findings pave the way to further studies aimed at investigating the BM microvasculature in presymptomatic *twitcher* mice. Notably, a lower rate of HSC engraftment has been observed in homozygous *twitcher* P8 recipients compared to heterozygous littermates [13], pointing to the presence of possible defects in the BM microvasculature also in presymptomatic animals. Further studies will be required to assess the impact of GALC deficiency on BM cell types other than SECs whose alteration may contribute to the decreased functionality of the hematopoietic niche [13,24].

Previous observations had shown that inhibition of VEGFR2 signaling in sublethally and lethally irradiated mice impairs SEC reconstruction and prevented hematopoietic stem and progenitor cell engraftment and the reconstitution of hematopoiesis following BM transfer [15]. Thus, our data suggest that morphofunctional defects of GALC-deficient BM SECs may contribute to the limited therapeutic efficacy of HSCT in KD patients, including those undergoing myeloablative HSCT, and identify BM SECs as a novel player in the pathogenesis of this disease.

## 4. Materials and Methods 

### 4.1. Animals

Breeder *twitcher* heterozygous mice (C57BL/6J, twi/+; Jackson Laboratories, ME, USA) were maintained under standard housing conditions. Animal handling protocols were in accordance with Italian institutional guidelines for animal care and use. *Twitcher* mutation was determined by polymerase chain reaction (PCR) on DNA extracted from clipped tails [35]. In all the experiments, littermate wild type (wt) and homozygous (twi/twi) animals were used at postnatal day P36.

### 4.2. Immunohistochemistry

Femurs were fixed in formalin for 8 h and then decalcified for 2 h in a 100 mM EDTA solution. Formalin-fixed, paraffin-embedded samples were sectioned at a thickness of 3.0 µm, dewaxed, hydrated, and stained with H&E or processed for immunohistochemistry with rat anti-mouse CD31 (Dianova, Hamburg, Germany) and rabbit anti-mouse VEGFR2 (Cell Signaling Technology, Danvers, MA, USA) antibodies. Positive signal was revealed by 3,3′-diaminibenzidine (Roche) staining. Sections were counterstained with Carazzi’s hematoxylin before analysis by light microscopy. Images were acquired with a Zeiss Axioplan 2 microscope and image analysis was carried out using the open-source ImageJ software.

### 4.3. Two-Photon Microscopy

#### 4.3.1. Femurs Collection and Bone Clearing

After euthanasia, mice were transcardially perfused with 0.01 M phosphate-buffered saline (PBS) (Sigma-Aldrich, Milan, Italy) followed by 4% paraformaldehyde (PFA) (VWR). After perfusion, femurs were harvested and post fixed overnight in 4% PFA. Bones were rinsed and incubated in PBS at 4.0 °C for 24 h. De-mineralization was conducted with 10% EDTA (Lonza, Basel, Switzerland) in 0.01 M PBS (pH 8.0) at 4 °C and EDTA buffer was exchanged daily for two weeks. Afterward, bones were immersed in the X-CLARITY polymerization solution composed by hydrogel solution and the polymerization initiator (Logos Biosystems, Villeneuve d’Ascq, France) and incubated for 24 h at 4.0 °C, polymerized for 3 h at a vacuum of −90 kPa and a temperature of 37.0 °C and rinsed with PBS. Then, samples were processed in the electrophoretic tissue clearing (ETC) solution (Logos Biosystems) for 16 h (checked every 4 h) at a current of 1.1A, temperature of 37.0 °C and pump speed of 30 rpm. Bones were then passively clarified at 37.0 °C for 3 weeks in ETC solution.

#### 4.3.2. Bone Immunostaining

After de-mineralization and clearing of the tissue, verified by its softness and optical transparency, femurs were rinsed with PBS for 24 h. Then, specimens were incubated for 48 h at 4.0 °C with rat anti-mouse CD31 antibody (Dianova, 1:100 dilution in PBS, 1% bovine serum albumin, 0.1% Tween 20) followed by 4 h incubation with AlexaFluor 594-conjugated secondary antibody (1:100, Thermo Fisher Scientific, Waltham, MA, USA). Then, samples were stored in PBS at 4.0 °C.

#### 4.3.3. Image Acquisition and Processing

For imaging, bones were held in 1% low melting agarose. Two-photon imaging was performed on a Zeiss LSM880 equipped with a EC Plan-Neofluar 20×/0.50 controlled by Zen Black 2 (Zeiss GmbH, Oberkochen, Germany). The excitation source was a Ti:Sapphire femtosecond laser cavity (Chameleon Vision II, Coherent), operating at 80 MHz and tuned to a wavelength of 750 nm for AlexaFluor 594 excitation and to a wavelength of 860 nm for second harmonic generation imaging of stromal collagen. To reconstruct large areas, 15% overlapping image tiles were acquired in Z-stack and processed using Zen Black 2 software, while 3D visualization was obtained using Zen Blue 2 (Zeiss GmbH).

### 4.4. Quantitative RT-PCR (RT-qPCR) Analysis

BM samples were collected and processed upon BM flushing. Total RNA was extracted from BM samples using TRIzol Reagent according to manufacturer’s instructions (Invitrogen, Carlsbad, CA, USA). Contaminating DNA was digested using DNAse (Promega, Madison, WI, USA) and 2.0 μg of total RNA were retro-transcribed with MMLV reverse transcriptase (Invitrogen) using random hexaprimers in a final 20 μL volume. Then, 1/20th of the reaction was analyzed for the expression of the indicated genes by RT-qPCR performed with a ViiATM7 Real-Time PCR Detection System (Applied Biosystems, Foster City, CA, USA) using a iQTM SYBR Green Supermix (Biorad, Hercules, CA, USA) according to manufacturer’s instructions. In each experiment, data were normalized for *Gapdh* expression and an arbitrary value equal to 1.0 was assigned to the levels of expression of the gene(s) measured in one wild type bone marrow sample that was used as reference. Primers used for RT-qPCR analysis are reported in Table 1. 

### 4.5. HUVEC Immunofluorescence Analysis

Confluent HUVECs seeded on gelatine-coated µ-slide 8 well chambers (Ibidi, Grafelfing, Germany) were treated overnight with 50 μM psychosine, fixed in 4% paraformaldehyde in PBS, permeabilized with 0.1% Triton-X100, and saturated with 3% BSA in PBS. Then, cells were incubated with anti-VE cadherin antibody. Actin cytoskeleton was visualized by incubation with rhodamine phalloidin and nuclei were counterstained with DAPI. Cells were photographed using a Zeiss Axiovert 200 M epifluorescence microscope equipped with Apotome and a Plan-Apochromat ×63/1.4 NA oil objective.

### 4.6. ELISA

The BM levels of VEGF-A and FGF2 were assessed by ELISA kits (mouse VEGF quantikine ELISA kit (R&D) and FGF2 ELISA kit (Cloud-Clone Corporation, Houston, TX, USA) according to the manufacturer’s instructions.

### 4.7. Statistical Analysis

Statistical analysis was performed using Prism 6 (GraphPad Software, San Diego, CA, USA). Student’s *t*-test for unpaired data (two-tailed) was used to test the probability of significant differences between two groups of samples. Differences were considered significant when *p* < 0.05.

## Figures and Tables

**Table 1:** Oligonucleotide primers used for RT-qPCR analysis.

Gene	Forward	Reverse
*Cd31*	5′-CGGTTATGATGATGTTTCTGGA-3′	5′-AAGGGAGGACACTTCCACTTCT-3′
*Vegfr2*	5′-AGATGCCCGACTCCCTTT-3′	5′-TTTCCCAGAGCAACACACC-3′
*Vegfa*	55′-ACCTCCACCATGCCAAGT-3′	5′-TCAATCGGACGGCAGTAG-3′
*Fgf2*	5′-CCTTCCCACCAGGCCACTTAA-3’	5′-GGTCCGTTTTGGATCCGAGTTT-3’
*Angpt1*	5′-TCATGCTAACAGGAGGTTGGT-3′	5′-ATGGTGGTGGAACGTAAGGA-3′
*Angpt2*	5′-GCTGGGCAATGAGTTTGTCT-3′	5′-CCTGGTTGGCTGATGCTACT-3′
*Il1α*	5′-CAGTTCTGCCATTGACCATC-3′	5′-GAATCTTCCCGTTGCTTGAC-3′
*Tnfα*	5′-GCCTCTTCTCATTCCTGCTT-3′	5′-TGATCTGAGTGTGAGGGTCTG-3′
*Tgfβ1*	5′-TTGCTTCAGCTCCACAGAGA-3′	5′-TACTGTGTGTCCAGGCTCCA-3′
*Gapdh*	5′-GAAGGTCGGTGTGAACGGATT-3′	5′-TGACTGTGCCGTTGAATTTG-3′

## Abbreviations

KD	Krabbe disease
CNS	central nervous system
SEC	sinusoidal endothelial cell
GALC	β-galactosylceramidase
HSC	hematopoietic stem cell
HSCT	HSC transplantation
BM	bone marrow
VEGFR2	vascular endothelial growth factor receptor-2
